# Non-Equilibrium Relations for Bounded Rational Decision-Making in Changing Environments

**DOI:** 10.3390/e20010001

**Published:** 2017-12-21

**Authors:** Jordi Grau-Moya, Matthias Krüger, Daniel A. Braun

**Affiliations:** 1 Max Planck Institute for Intelligent Systems, Stuttgart 70569, Germany; 2 Max Planck Institute for Biological Cybernetics, Tübingen 72076, Germany; 3 PROWLER.io, Cambridge CB2 1LA, UK; 4 4th Institute for Theoretical Physics, Universität Stuttgart, Stuttgart 70569, Germany; 5 Institute of Neural Information Processing, Universität Ulm, Ulm 89081, Germany

**Keywords:** free energy, bounded rationality, anticipation, adaptation

## Abstract

Living organisms from single cells to humans need to adapt continuously to respond to changes in their environment. The process of behavioural adaptation can be thought of as improving decision-making performance according to some utility function. Here, we consider an abstract model of organisms as decision-makers with limited information-processing resources that trade off between maximization of utility and computational costs measured by a relative entropy, in a similar fashion to thermodynamic systems undergoing isothermal transformations. Such systems minimize the free energy to reach equilibrium states that balance internal energy and entropic cost. When there is a fast change in the environment, these systems evolve in a non-equilibrium fashion because they are unable to follow the path of equilibrium distributions. Here, we apply concepts from non-equilibrium thermodynamics to characterize decision-makers that adapt to changing environments under the assumption that the temporal evolution of the utility function is externally driven and does not depend on the decision-maker’s action. This allows one to quantify performance loss due to imperfect adaptation in a general manner and, additionally, to find relations for decision-making similar to Crooks’ fluctuation theorem and Jarzynski’s equality. We provide simulations of several exemplary decision and inference problems in the discrete and continuous domains to illustrate the new relations.

## 1. Introduction

A number of recent studies has pointed out mathematical equivalences between thermodynamic systems described by statistical mechanics and information processing systems [1,2,3,4]. In particular, it has been suggested that decision-makers with constrained information-processing resources can be described in analogy to closed physical systems in contact with a heat bath that seek to minimize energy [1]. In this analogy, decision-makers can be thought to act in a way that minimizes a cost function or, equivalently, that maximizes a utility function in lieu of an energy function. Classic decision theory [5,6] states that, given a set of actions X and a set of observations O, the perfectly rational decision-maker should choose the best possible action $x^{*}\in X$ that maximizes the expected utility U(x):

(1) $$x^{*}=\underset{x}{\mathrm{argmax}}U\left(x\right)=\underset{x}{\mathrm{argmax}}\sum_{o\in O}p\left(o|x\right)V\left(o\right),$$

where p(o|x) is the probability of the outcome *o* given action *x* and V(o) indicates the utility of this outcome. However, maximizing the expected utility is in general a costly computational operation that real decision-makers might not be able to perform.

Decision-makers that are unable to choose the best possible action $x^{*}$ due to a lack of computational resources have traditionally been studied in the field of bounded rationality. Originally proposed by Herbert Simon [7,8], bounded rationality comprises a medley of approaches ranging from optimization-based approaches like bounded optimality (searching for the program that achieves the best utility performance on a particular platform) [9,10,11] and meta-reasoning (optimizing the cost of reasoning) [12,13,14] to heuristic approaches that reject the notion of optimization [15,16,17]. Recently, new impulses for the development of bounded rationality theory have come from information-theoretic and thermodynamic perspectives on the general organization of perception-action-systems [1,3,18,19,20,21,22,23,24,25,26,27]. In the economic and game-theoretic literature, these models have precursors that have studied bounded rationality inspired by stochastic choice rules originally proposed by Luce, McFadden and others [2,28,29,30,31,32,33,34,35,36,37,38,39]. In most of these models, decision-makers face a trade-off between the attainment of maximum utility and the required information-processing cost measured as an entropy or relative entropy. The optimal solution to this trade-off usually takes the form of a Boltzmann-like distribution analogous to equilibrium distributions in statistical physics. The decision-making process can then be conceptualized as a change from a prior strategy distribution to a posterior strategy distribution, where the change is triggered by a change in the utility landscape. However, studying changes in equilibrium distributions neglects not only the time required for this change, but also the adaptation process itself.

The main contribution of this paper is to show that the analogy between equilibrium thermodynamics and bounded-rational decision-making [1] can be extended to the non-equilibrium domain under the assumption that the temporal evolution of the utility function is externally driven and does not depend on the decision-maker’s action. This allows for new predictions that can be tested in experimental setups investigating decision-makers that choose between multiple alternatives. When given sufficient time to adjust to the problem such a decision-maker may achieve a bounded optimal performance given the available precision, which may be described by an equilibrium distribution; for example, a dart thrower that has fully adapted her/his personal best performance after extensive training with prism glasses. However, if given insufficient time, the decision-maker may not achieve bounded optimal performance, but only an inferior performance biased by the specific information-processing mechanisms used by the decision-maker, which may in general be described by a non-equilibrium distribution; for example, a dart thrower that is wearing prism glasses for the first time and plays according to a non-adaptive strategy thereby “dissipating” utility. The connection between the non-equilibrium and equilibrium domains is tied with the concept of dissipation and its role in fluctuation theorems, which are important recent results in non-equilibrium thermodynamics.

The paper is organized as follows. In Section 2, we recapitulate the relation between bounded rational decision-making and equilibrium thermodynamics. In Section 3, we relate decision-making processes to non-equilibrium thermodynamics. In Section 4, we generalize concepts from non-equilibrium thermodynamics to make them applicable to a wider range of decision-making problems. In particular, we include a derivation of a generalized Jarzynski equality and a generalized Crooks’ theorem for decision-making. We provide simulations to illustrate the new relations in different decision-making scenarios. In Section 5, we discuss our results.

## 2. Equilibrium Thermodynamics and Decision-Making

In thermodynamics, closed physical systems in thermal equilibrium with their environment are described by equilibrium distributions that do not change over time. For example, a gas in a box distributes its particles evenly over the entire space and will stay this way and not spontaneously concentrate in a corner of the box. When changing constraints of the physical system, equilibrium thermodynamics allows predicting the final state after the change has taken place. For example, when opening a divider between two boxes, the gas will expand further until it fills the entire space evenly. This way, equilibrium thermodynamics allows describing system behaviour as a change from a prior equilibrium distribution to a posterior equilibrium distribution triggered by a change in external constraints.

On an abstract level, one can think about changes in the distribution of a random variable from a prior to a posterior distribution as the basis of information-processing. In Bayesian inference, for example, we update current prior beliefs $p_{0}\left(x\right)$ by means of a likelihood to obtain a posterior belief $p_{1}\left(x\right)$. Similarly, decision-making can be regarded as a process of changing a prior strategy $p_{0}\left(x\right)$ to a posterior strategy $p_{1}\left(x\right)$ through a process of deliberation [1], thereby emphasizing the stochastic nature of choice [40]. According to [1], such transitions from prior to posterior with information constraints can be formalized by optimizing the variational problem:

(2) $$p_{1}^{\mathrm{eq}}\left(x\right)=\underset{p}{\mathrm{argmax}}\;\Delta F\left[p\right]$$

where:

(3) $$\Delta F\left[p\right]:=\sum_{x}p\left(x\right)\Delta U\left(x\right)-\frac{1}{\beta }D_{\mathrm{KL}}\left(p\right\|p_{0}),$$

is a free energy functional, ΔU(x) is a change in utility (analogous to the notion of gains and losses in prospect theory [15]), $D_{\mathrm{KL}}\left(\cdot \|\cdot \right)$ is the Kullback–Leibler divergence or relative entropy and β is a real-valued parameter that translates from informational units into utility units. Accordingly, Equation (3) optimizes a trade-off between utility gains and information-processing resources quantified by the “information distance” between prior and posterior. In a physical system (where the energy function corresponds to a negative utility), Equation (3) evaluated at the optimum $p_{1}^{\mathrm{eq}}$ quantifies the negative free energy difference $\Delta F[p_{1}^{\mathrm{eq}}]$ between the final state 1 and the initial state 0 assuming an isothermal process with respect to the inverse temperature β and a negative energy difference of $\Delta U=U_{1}-U_{0}$.

For a given information cost parameter β, the bounded rational decision-maker optimally trades off utility gain against informational resources according to Equation (2), thereby following the strategy:

(4) $$p_{1}^{\mathrm{eq}}\left(x\right)=\frac{1}{Z_{\beta }}p_{0}\left(x\right)e^{\beta \Delta U(x)}$$

with partition function $Z_{\beta }=\sum_{x}p_{0}\left(x\right)e^{\beta \Delta U(x)}$. When inserting the optimal strategy $p_{1}^{\mathrm{eq}}\left(x\right)$ into Equation (3), the certainty-equivalent value of strategy $p_{1}^{\mathrm{eq}}$ is determined by

(5) $$\Delta F^{\mathrm{eq}}:=\Delta F\left[p_{1}^{\mathrm{eq}}\right]=\frac{1}{\beta }\log Z_{\beta }.$$

For β→0, the cost of computation dominates, and the optimal strategy is given by the prior strategy $p_{1}^{\mathrm{eq}}\left(x\right)=p_{0}\left(x\right)$ with the value $\lim_{\beta \to 0}\Delta F\left[p_{1}^{\mathrm{eq}}\right]={\langle \Delta U(x)\rangle }_{p_{0}\left(x\right)}$. This models a decision-maker that cannot afford any information-processing. When information costs are low (β→∞), the optimal strategy $p_{1}^{\mathrm{eq}}\left(x\right)$ places all the probability mass on the maximum of ΔU(x), and the value of the strategy is $\lim_{\beta \to \infty }\Delta F\left[p_{1}^{\mathrm{eq}}\right]=\max_{x}\Delta U\left(x\right)$. This models a perfectly rational decision-maker that can hand pick the best action. While this model includes maximum (expected) utility decision-making of Equation (1) as a special case, note that conceptually, the formulation of the decision problem as a variational problem in the probability distribution is very different from traditional approaches that define an optimization problem directly in the space of actions.

One possible objection to the strategy (4) is that it requires computing the partition sum $Z_{\beta }$ over all possible actions, which is in general an intractable operation; even though Equation (4) could still be of descriptive value. It should be noted, however, that the decision-maker is not required to explicitly compute $p_{1}^{\mathrm{eq}}\left(x\right)$; it suffices to produce a sample from $p_{1}^{\mathrm{eq}}\left(x\right)$ to generate a decision. This can be achieved, for example, by Markov Chain Monte Carlo (MCMC) methods that are specifically designed to avoid the explicit computation of partition sums [41]. In the following, we recapitulate two simple MCMC examples in the context of decision-making: a bounded rational decision-maker that uses a rejection sampling scheme and a bounded rational decision-maker that uses a variant of the Metropolis–Hastings scheme [42].

### Exemplary Bounded Rational Decision-Makers

The optimal distribution (4) can be implemented, for example, by a decision-maker that follows a probabilistic satisficing strategy with aspiration level $T\geq \max_{x}\Delta U\left(x\right)$. Such a decision-maker optimizes the utility ΔU(x) by drawing samples from the prior distribution $x_{s}∼p_{0}\left(x\right)$ and accepts with certainty the first sample $x_{s}$ with utility $\Delta U(x_{s})\geq T$ reaching the aspiration level *T* or any sample with utility below the aspiration level with acceptance probability $p_{\mathrm{accept}}=\exp\left(\beta \left(\Delta U\left(x_{s}\right)-T\right)\right)$. The most efficient samplers use $T=\max_{x}\Delta U\left(x\right)$. For samplers with $T>\max_{x}\Delta U\left(x\right)$, the probability distribution (4) is still recovered, but more samples are required, as the acceptance probability $p_{\mathrm{accept}}$ is decreased in this case. This strategy is a particular version of the rejection sampling algorithm and is shown in pseudo-code in Algorithm 1. We can see the direct connection between informational resources (“distance away from the prior”) and the average number of samples required until acceptance, as the expected number of required samples from $p_{0}$ to obtain one accepted sample from $p_{1}^{\mathrm{eq}}$ is given by ${\bar{n}}_{\beta }=\exp\left(\beta T\right)/Z_{\beta }\geq \exp D_{\mathrm{KL}}\left(p\|p_{0}\right)$ [43]. In the limit of zero information-processing with $D_{\mathrm{KL}}\left(p\|p_{0}\right)=0$ in the high-cost regime β→0, the sampling complexity tends to its minimum ${\bar{n}}_{\beta \to 0}\to 1$.

**Algorithm 1** Rejection sampling.
**repeat**    $x∼p_{0}\left(x\right)$    u∼Uniform[0,1]    **if** u≤exp(β(ΔU(x)−T)) **then** accept**until** accept**return** *x*


In case we do not want to set an absolute aspiration level *T*, an incremental version of such a decision-maker can be realized by the Metropolis–Hastings scheme. Given a current action proposal *x*, the decision-maker generates a novel proposal $x^{\prime }$ from $p_{0}\left(x\right)$. If $\Delta U\left(x^{\prime }\right)\geq \Delta U\left(x\right)$, then the sample is accepted with certainty. An inferior sample is accepted with probability $p_{\mathrm{accept}}=\exp(\beta \left(\Delta U\left(x^{\prime }\right)-\Delta U\left(x\right)\right)$. The aspiration level in this case is variable and always given by the utility of the previous sample. This corresponds to a Markov chain with transition probability $p\left(x^{\prime }|x\right)=p_{0}\left(x^{\prime }\right)\min\left\{1,\exp\left(\beta \left(\Delta U\left(x^{\prime }\right)-\Delta U\left(x\right)\right)\right)\right\}$ and stationary distribution $p_{1}^{\mathrm{eq}}\left(x\right)$. This Markov chain fulfils detailed balance, i.e., $p_{1}^{\mathrm{eq}}\left(x\right)p\left(x^{\prime }|x\right)=p_{1}^{\mathrm{eq}}\left(x^{\prime }\right)p\left(x|x^{\prime }\right)$, which implies that after infinitely many repetitions, the samples *x* will follow the stationary distribution. This Markov chain is a particular version of the Metropolis–Hastings algorithm and is shown in pseudo-code in Algorithm 2. The longer the chain runs, the further the distribution of *x* will move away from the prior, i.e., the higher the informational resources will be. Finally, the chain reaches the equilibrium distribution.

**Algorithm 2** Metropolis–Hastings sampling.
$x∼p_{0}\left(x\right)$  **repeat**     $x^{\prime }∼p_{0}\left(x^{\prime }\right)$      u∼Uniform[0,1]      **if** $u\leq \exp\left(\beta \left(\Delta U\left(x^{\prime }\right)-\Delta U\left(x\right)\right)\right)$ **then** accept $x\leftarrow x^{\prime }$  **until** chain has converged to equilibrium**return** *x*


## 3. Non-Equilibrium Thermodynamics and Decision-Making

If decision-making is emulated by a Markov chain that converges to an equilibrium distribution and one wants to be absolutely certain that the chain has reached equilibrium, then one has to wait for an infinitely long time. For finite times, when considering only a limited number of samples from the chain, we are dealing in general with non-equilibrium any time process models, i.e., computational processes that can be interrupted at any time to deliver an answer; a representative example being the Metropolis–Hastings dynamics when Algorithm 2 is run for k∈N steps. The same holds true for a rejection sampling decision-maker. Even though Algorithm 1 generates equilibrium samples with a finite expected number of samples ${\bar{n}}_{\beta }$, before running the algorithm, it is unknown whether after a particular number of steps *k*, a sample will be accepted or not; to have certainty, we would have to allow for an infinite amount of time (k→∞). In an any time version of rejection sampling, the probability of not accepting a sample after *k* tries is given by $q_{k}={\left[1-Z(\beta )\exp(-\beta T)\right]}^{k}$, in which case the sample $x_{s}$ will be distributed according to the prior distribution $p_{0}\left(x\right)$. The probability of accepting a sample that is distributed according to $p_{1}^{\mathrm{eq}}\left(x\right)$ after *k* tries is given by $1-q_{k}$. Accordingly, the action at time *k* is a mixture distribution of the form:

(6) $$p_{k}^{\mathrm{neq}}\left(x\right)=\left(1-q_{k}\right)p_{1}^{\mathrm{eq}}\left(x\right)+q_{k}p_{0}\left(x\right).$$

The distribution $p_{k}^{\mathrm{neq}}\left(x\right)$ is a non-equilibrium distribution that reaches equilibrium $p_{k}^{\mathrm{neq}}\left(x\right)\to \;p_{1}^{\mathrm{eq}}\left(x\right)$ for k→∞. In the following, we ask how far the tools of non-equilibrium thermodynamics are applicable to such any time decision-making processes.

### 3.1. Non-Equilibrium Thermodynamics

In thermodynamics, non-equilibrium processes are often modelled in the presence of an external parameter λ(t)∈[0,1] that determines how the energy function $E_{\lambda }\left(x\right)$ changes over time; for example, when switching on a potential in a linear fashion, the energy would be $E_{\lambda }\left(x\right)=E_{0}\left(x\right)+\;\lambda \;\left(E_{1}\left(x\right)-E_{0}\left(x\right)\right)$. When the change in the parameter λ is done infinitely slowly (quasi-statically), the system’s probability distribution follows exactly the path of equilibrium distributions (for any λ) $p_{\lambda }\left(x\right)=\frac{1}{Z_{\lambda }}e^{-\beta E_{\lambda }\left(x\right)}$. Importantly, when the switching of the external parameter λ is done in finite time, the trajectory in phase space of the evolving thermodynamic system can potentially be very different from the quasi-static case. In particular, the non-equilibrium path of probability distributions is going to be, in general, different from the equilibrium path. We define the trajectory of an evolving system as a finite sequence of states $x:=(x_{0},x_{1},\cdots x_{N})$ at times $t_{0},t_{1},\ldots ,t_{N}$, and the probability of the trajectory as $p\left(x\right):=p\left(x_{0}|t_{0}\right)\prod_{n=1}^{N}p\left(x_{n}|x_{n-1},t_{n}\right)$ that follows Markovian dynamics. Since λ is then a function of time $\lambda (t_{n})$, we can effectively consider the energy as a function of state and time $E\left(x_{n},t_{n}\right):=E_{\lambda (t_{n})}\left(x_{n}\right)$. Accordingly, the internal energy of the system can change in two ways depending on changes in the two variables $t_{n}$ and $x_{n}$. Assuming discrete time steps, an energy change due to a change in the external parameter is defined as the work [24,44]:

$$w\left(x_{n-1},t_{n-1}\to t_{n}\right)=E\left(x_{n-1},t_{n}\right)-E\left(x_{n-1},t_{n-1}\right)$$

and an energy change due to an internal state change is defined as the heat [24,44]:

$$q\left(x_{n-1}\to x_{n},t_{n}\right)=E\left(x_{n},t_{n}\right)-E\left(x_{n-1},t_{n}\right).$$

For an entire process trajectory $x_{0},x_{1},\ldots ,x_{N}$ measured at times $t_{0},t_{1},\ldots ,t_{N}$, the extracted work is $W\left(x\right)=-\sum_{n=1}^{N}w\left(x_{n-1},t_{n-1}\to t_{n}\right)$, and the heat transferred to the environment by relaxation steps is $Q\left(x\right)=-\sum_{n=1}^{N}q\left(x_{n-1}\to x_{n},t_{n}\right)$. The sum of work and heat is the total energy difference $\Delta E\left(x\right):=-\left(E\left(x_{N},t_{N}\right)-E\left(x_{0},t_{0}\right)\right)=W\left(x\right)+Q\left(x\right)$. In expectation with respect to p(x), we define the average work $W:={\langle W(x)\rangle }_{p(x)}$, the average heat $Q:={\langle Q(x)\rangle }_{p(x)}$ and the average energy change $\Delta E:={\langle \Delta E(x)\rangle }_{p(x)}$. With these averaged quantities, we obtain the first law of thermodynamics in its usual form:

(7) $$\begin{array}{cc} \Delta E & =W+Q\\ & =W+T\Delta S+W^{\mathrm{diss}}. \end{array}$$

The heat *Q* can be decomposed into a reversible and an irreversible part given by the entropy difference $\Delta S=-(S\left(t_{N}\right)-S\left(t_{0}\right))$, which is multiplied by the temperature *T* and the average dissipation $W^{\mathrm{diss}}$. The concept of dissipation will be particularly useful later to quantify inefficacies in decision-making processes with limited time. By identifying the equilibrium free energy difference with $\Delta F:=-(F\left(t_{N}\right)-F\left(t_{0}\right))=\Delta E-T\Delta S$, we can then write the first law as:

(8) $$W=\Delta F-W^{\mathrm{diss}}.$$

In case of a quasi-static process, the extracted work *W* exactly coincides with the equilibrium free energy difference (thus, $W^{\mathrm{diss}}=0$). In the case of a finite time process, we can express the average dissipated work as [45,46,47]:

(9) $$W^{\mathrm{diss}}:={\langle W^{\mathrm{diss}}\left(x\right)\rangle }_{p(x)}=\Delta F-W=\frac{1}{\beta }D_{\mathrm{KL}}\left(p\left(x\right)\|p^{†}\left(x\right)\right)$$

where $D_{\mathrm{KL}}$ is the relative entropy that measures in bits the distinguishability between the probability of the forward in time trajectory p(x) and the probability of the backward in time trajectory $p^{†}\left(x\right):=p\left(x_{N}|t_{N}\right)\prod_{n=1}^{N}p\left(x_{n-1}|x_{n},t_{n-1}\right)$. From the positivity of the relative entropy, we can immediately see the non-negativity of entropy production $W^{\mathrm{diss}}\geq 0$, which allows stating the second law of thermodynamics in the form:

(10) W≤ΔF.

#### 3.1.1. Crooks’ Fluctuation Theorem

Equation (9) can be given in a more general form without averages. It is possible to relate the reversibility of a process with its dissipation at the trajectory level. Given a protocol $\Lambda =(\lambda_{0},\lambda_{1},\cdots \lambda_{N})$, i.e., a sequence of external parameters, the probability p(x) of observing a trajectory of the system in phase space compared with its time-reversal conjugate $p^{†}\left(x\right)$ (when using the time-reversal protocol $\Lambda^{†}=\left(\lambda_{N},\lambda_{N-1},\cdots \lambda_{0}\right)$) depends on the dissipation of the trajectory in the forward direction according to the following expression:

$$\frac{p(x)}{p^{†}\left(x\right)}=e^{\beta W^{\mathrm{diss}}\left(x\right)}\;,$$

where $W^{\mathrm{diss}}\left(x\right)=\Delta F-W\left(x\right)$ is the dissipated work of the trajectory. For this relation to be true, both backward and forward processes must start with the system in equilibrium. Intuitively, this means that the more the entropy production (measured by the dissipated work), the more distinguishable are the trajectories of the forward protocol compared to the backward protocol.

#### 3.1.2. Jarzynski Equality

Additionally, another relation of interest in non-equilibrium thermodynamics has recently been found transforming the inequality of Equation (10) into an equality, the so-called Jarzynski equality [48]:

(11) $${\langle e^{\beta W(x)}\rangle }_{p(x)}=e^{\beta \Delta F}$$

where the angle brackets denote an average over all possible trajectories x of a process that drives the system from an equilibrium state at λ=0 to another state at λ=1. Specifically, the above equality says that, no matter how the driving process is implemented, we can determine equilibrium quantities from work fluctuations in the non-equilibrium process; or in other words, this equality connects non-equilibrium thermodynamics with equilibrium thermodynamics. In the following, we are interested in the question whether there exist similar relations such as the Jarzynski equality or Crooks’ fluctuation theorem and similar underlying concepts such as dissipation and time reversibility for the case of decision-making.

### 3.2. Non-Equilibrium Thermodynamics Applied to Bounded Rational Decision-Making

In direct analogy to the previous section, in the following, we consider decision-makers faced with the problem of optimizing a changing utility function. We assume that time is discretized into *N* steps $t_{0},\ldots ,t_{N}$. For each time step $t_{n}$, the utility is assumed to be constant, but it can change between time steps, such that we have a sequence of decision problems expressed by the changes in utility $\Delta U\left(x,t_{0}\to t_{1}\right),\cdots ,\Delta U\left(x,t_{N-1}\to t_{N}\right)$. At each time point $t_{n}$, the decision-maker chooses action $x_{n}$, such that we can summarize the decision-maker’s choices by a vector $x:=(x_{0},\ldots ,x_{N})$. The behaviour of the decision-maker is characterized by the probability $p\left(x\right):=p\left(x_{0}|t_{0}\right)\prod_{n=1}^{N}p\left(x_{n}|x_{n-1},t_{n}\right)$ with $p\left(x_{0}|t_{0}\right)=p_{0}\left(x_{0}\right)$, assuming that the initial strategy is a bounded rational equilibrium strategy. In this setup, we assume that the changes in the utility function are externally driven, i.e., the decision-maker’s actions cannot change the temporal evolution of the utility function. Furthermore, note that the decision-maker does not know how the utility changes over time. Accordingly, the best the decision-maker can do is to optimize the current utility as much as possible.

At time $t_{0}$, the decision-maker starts with selecting an action $x_{0}$ from the distribution $p(x_{0}|t_{0})$ and the utility changes instantly by $\Delta U(x,t_{0}\to t_{1})$. The decision-maker can then adapt to this utility change with the distribution $p(x_{1}|x_{0},t_{1})$ and select the action $x_{1}$ at time $t_{1}$, but at this point, the utility is already changing again by $\Delta U(x,t_{1}\to t_{2})$. The adaptation from $p(x_{0}|t_{0})$ to $p(x_{1}|x_{0},t_{1})$ is analogous to a physical relaxation process and implies a strategy change between $x_{0}$ and $x_{1}$. In general, at each time point $t_{n-1}$, the decision-maker chooses action $x_{n-1}$ while the current utility changes by:

$$\Delta U\left(x_{n-1},t_{n-1}\to t_{n}\right)=U\left(x_{n-1},t_{n}\right)-U\left(x_{n-1},t_{n-1}\right).$$

This way, the decision-maker is always lagging behind the changes in utility, just like a physical system would lag behind the changes in the energy function. The utility $\Delta U(x_{n-1},t_{n-1}\to t_{n})$ gained by the decision-maker at time point $t_{n-1}$ parallels the concept of work in physics. For a whole trajectory, we define the total utility gain due to changes in the environment as $U\left(x\right)=\sum_{n=1}^{N}\Delta U\left(x_{n-1},t_{n-1}\to t_{n}\right)$. Note that the last decision $x_{N}$ can be ignored in this notation, as it does not contribute to the utility.

**Figure 1:**
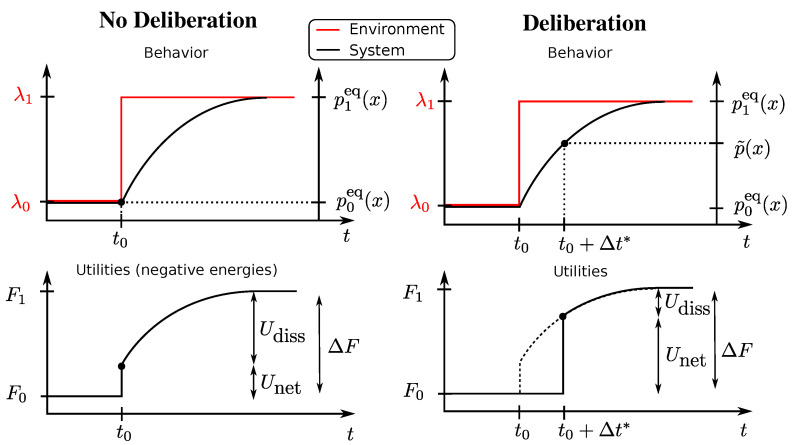
Temporal structure of the one-step decision problem. An instantaneous change in the environment occurs at time $t_{0}$ represented by a vertical jump from $\lambda_{0}$ to $\lambda_{1}$ in the upper panels that translates directly into a change in free energy difference represented by ΔF in the lower panels. The system’s previous state at $t_{0}$ is given by $p_{0}^{\mathrm{eq}}\left(x\right)$, i.e., the equilibrium distribution for $U_{\lambda_{0}}\left(x\right)$. The new posterior equilibrium is given by $p_{1}^{\mathrm{eq}}\left(x\right)$, i.e., the equilibrium distribution for $U_{\lambda_{1}}\left(x\right)$. When given unlimited time, the decision-maker will eventually evolve to $p_{1}^{\mathrm{eq}}\left(x\right)$. Deliberative and non-deliberative decision-makers differ in how much time they get to adapt to the change in utility before they have to choose an action *x* that provides them with the utility gain $\Delta U\left(x\right)=U_{\lambda_{1}}\left(x\right)-U_{\lambda_{0}}\left(x\right)$. Left: In direct analogy to physical thermodynamics, the non-deliberative decision-maker has to emit an action before it can adapt to any changes in utility and therefore acts according to the previous strategy $p_{0}^{\mathrm{eq}}\left(x\right)$ at time $t_{0}$. On average, with such a strategy, the utility gained is $U^{\mathrm{net}}=\sum_{x}p_{0}^{\mathrm{eq}}\left(x\right)\Delta U\left(x\right)$ at $t_{0}$ and the dissipation is $U^{\mathrm{diss}}=\Delta F-U^{\mathrm{net}}$. Right: The deliberative decision-maker is allowed to adapt to the change in utility for a certain time $\Delta t^{*}$ before the action has to be emitted. This deliberation period allows the decision-maker to compute a better strategy $\tilde{p}\left(x\right)$. In this case, the net utility is $U^{\mathrm{net}}=\sum_{x}\tilde{p}\left(x\right)\Delta U-\frac{1}{\beta }D_{\mathrm{KL}}\left(\tilde{p}\left(x\right)\left|\right|p_{0}^{\mathrm{eq}}\left(x\right)\right)$.

In Figure 1 (left column), we illustrate the setup for a one-step decision problem $\Delta U(x,t_{0}\to t_{1})$ with behaviour vector $x=(x_{0},x_{1})$. An instantaneous change in the environment occurs at time $t_{0}$ represented by a vertical jump from $\lambda_{0}$ to $\lambda_{1}$ in the upper panels that translates directly into a change in free energy difference represented by ΔF in the lower panels. The system’s previous state at $t_{0}$ is given by $p_{0}^{\mathrm{eq}}\left(x\right)$, i.e., the equilibrium distribution for $U_{0}$. The new equilibrium is given by $p_{1}^{\mathrm{eq}}\left(x\right)$, i.e., the equilibrium distribution for $U_{1}$. In this case, the behaviour vector is $x=(x_{0},x_{1})$ with $x_{0}∼p_{0}^{\mathrm{eq}}\left(x\right)$, and $x_{1}$ is ignored.

Similarly to Equation (8), we can now formulate the first law for decision-making as:

$$U=\Delta F-U^{\mathrm{diss}}$$

stating that the total average utility $U:={\langle U(x)\rangle }_{p(x)}$ is the difference between the bounded optimal utility (following the equilibrium strategy with precision β) expressed by the equilibrium free energy difference ΔF and the dissipated utility $U^{\mathrm{diss}}$. The dissipation for a trajectory $U^{\mathrm{diss}}\left(x\right):=\Delta F-U\left(x\right)$ measures the amount of utility loss due to the inability of the decision-maker to act according to the equilibrium distribution. This is because the decision-maker cannot anticipate the changes in the environment. At most, the decision-maker could act according to the equilibrium distributions of the previous environment. Thus, even with full adaptation, the decision-maker will always lag behind one time step and will therefore always dissipate.

Due to an equivalent version of Equation (9), we can also state the second law for decision-making $U^{\mathrm{diss}}\geq 0$, which implies that a purely adaptive decision-maker can gain a maximum utility that cannot be larger than the free energy difference:

U≤ΔF.

Similarly, we can obtain equivalent relationships to the Crooks fluctuation theorem:

(12) $$\frac{p(x)}{p^{†}\left(x\right)}=e^{\beta U^{\mathrm{diss}}\left(x\right)}\;,$$

and the Jarzynski equality:

(13) $${\langle e^{\beta U(x)}\rangle }_{p(x)}=e^{\beta \Delta F}$$

which both have the same implications as in the physical scenario and can be derived in the same way as in the physical counterpart [44]. In summary, we can say that an adaptive decision-maker, which has to act without knowing that the utility function has changed, follows the same laws as a thermodynamic physical system that is lagging behind the equilibrium.

### 3.3. Examples

In this section, we illustrate the applicability of thermodynamic non-equilibrium concepts in a series of simulations for different decision-making scenarios. In particular, we study two model classes: the first one contains simple one-step lag models of adaptation where equilibrium is always reached with one time step delay, and the second one contains more complex models of adaptation that do not necessarily equilibrate after one time step. In the first model class, we can easily study the relation between dissipation and the rate of information-processing, whereas in the second class of models, we can study more complex non-equilibrium phenomena such as learning hysteresis.

#### 3.3.1. One-Step Lag Models of Adaptation

Consider a learner that is adapted to their environment such that their behaviour can be described by the equilibrium distribution $p_{0}\left(x\right)$. For this idealized scenario, we assume that the learner can adapt their behaviour to any environment perfectly after a time lapse of Δt. This also means that before the lapse of Δt, the learner continues to follow their old strategy and is inefficient during this time span. We now consider two scenarios: first, where the environment changes suddenly by ΔU(x), and second, where the environment changes slowly in *N* small steps of ΔU(x)/N. In the first case, the learner is going to dissipate the utility:

$$U^{\mathrm{diss}}=\frac{1}{\beta }D_{\mathrm{KL}}\left(p_{0}\left(x\right)\|p_{1}^{\mathrm{eq}}\left(x\right)\right),$$

in the first time step. In all subsequent time steps, no more utility is wasted, assuming the environment does not change any more. In the second case, the utility function can be written as $U_{t}\left(x\right)=U_{0}\left(x\right)+\;\frac{t}{N}\Delta U\left(x\right)$ for t∈N:0≤t≤N. To compute the dissipated utility, we need to compare the learner’s behaviour in time step *t* to the bounded optimal behaviour, which is:

$$p^{\mathrm{eq}}\left(x,t\right)=\frac{1}{Z}p^{\mathrm{eq}}\left(x,t-1\right)e^{\frac{\beta }{N}\Delta U\left(x\right)}$$

for t>0. The overall average dissipated utility for the whole process is then

$$U_{N}^{\mathrm{diss}}=\frac{1}{\beta }\sum_{t=1}^{N}D_{\mathrm{KL}}\left(p^{\mathrm{eq}}\left(x,t-1\right)\|p^{\mathrm{eq}}\left(x,t\right)\right).$$

The net utility gain for the N-step scenario is $U_{N}^{\mathrm{net}}=\Delta F-U_{N}^{\mathrm{diss}}$. Note that:

$$U_{N}^{\mathrm{diss}}\geq U_{N+1}^{\mathrm{diss}}$$

and consequently, in direct analogy to a quasi-static change in a thermodynamic system, we get vanishing dissipation ($U_{N}^{\mathrm{diss}}\to 0$) if the utility changes infinitely slowly (N→∞ and ΔU(x)/N→0), such that the net utility equals the free energy difference $U_{N}^{\mathrm{net}}=\Delta F$.

#### 3.3.2. Bayesian Inference as a One-Step Lag Process

Bayesian inference mechanisms naturally have step by step dynamics that update beliefs with new incoming observations. Again, we can consider two scenarios: first where the learner updates their belief abruptly by processing a huge chunk of data in one go, and second, where belief updates are incremental with small chunks of data at each time step. Here, we show how the size of the chunks of data affect the overall surprise of the decision-maker and how this relates to dissipation applying the free energy principle to Bayesian inference.

Traditionally, Bayes’ rule is obtained directly from the product rule of probabilities p(θ,D)=p(θ)p(D|θ)=p(D)p(θ|D) where θ correspond to the different available hypotheses and D corresponds to the dataset. However, Bayes’ rule can also be considered to be a consequence of the maximization of the free energy difference with the log-likelihood as a utility function [49,50,51]. In this view, the posterior belief p(θ|D) is a trade-off between maximizing the likelihood p(D|θ) and minimizing the distance from the prior $p_{0}\left(\theta \right)$ such that:

(14) $$\begin{array}{cc} p(\theta |D) & =\underset{\tilde{p}}{\mathrm{argmax}}\Delta F\left[\tilde{p}\right]=\underset{\tilde{p}}{\mathrm{argmax}}\int \tilde{p}\left(\theta |D\right)\log p\left(D|\theta \right)d\theta -\frac{1}{\beta }\int \tilde{p}\left(\theta |D\right)\log\frac{\tilde{p}\left(\theta |D\right)}{p_{0}\left(\theta \right)}d\theta \end{array}$$

(15) $$\begin{array}{c} =\frac{1}{Z}p_{0}\left(\theta \right)e^{\beta \log p(D|\theta )}=\frac{1}{Z}p_{0}\left(\theta \right)p{\left(D|\theta \right)}^{\beta } \end{array}$$

is identical to Bayes’ rule when β=1. For β→∞, we recover the maximum likelihood estimation method as the density update is $p\left(\theta |D\right)=\delta \left(\theta -\theta_{\mathrm{MLE}}\right)$ with $\theta_{\mathrm{MLE}}={\mathrm{argmax}}_{\theta }\log p\left(D|\theta \right)$.

Such a Bayesian learner with prior $p_{0}\left(\theta \right)$ that incorporates all the data *X* at once is going to experience the expected surprise $S=-\int p_{0}\left(\theta \right)\log p\left(D|\theta \right)d\theta$. In contrast, a Bayesian learner that incorporates the data slowly in *N* steps (thus, the dataset $D=(X_{1},\cdots ,X_{N})$ is divided in *N* parts) experiences an expected surprise of $S=-\sum_{n=1}^{N}\int p\left(\theta |X_{1},\cdots ,X_{n-1}\right)\log p\left(X_{n}|\theta \right)d\theta$. Here, the surprise S corresponds to the thermodynamic concept of work. The first law can then be written as:

$$\Delta F+S=U^{\mathrm{diss}}$$

where the equivalent of dissipation corresponds to:

$$U^{\mathrm{diss}}=\frac{1}{\beta }D_{\mathrm{KL}}(p_{0}\left(\theta \right)\|p_{\mathrm{eq}}\left(\theta |D\right)).$$

when processing all the data at once and to:

$$U^{\mathrm{diss}}=\frac{1}{\beta }\sum_{n=1}^{N}D_{\mathrm{KL}}(p\left(\theta |X_{<n}\right)\|p_{\mathrm{eq}}\left(\theta |X_{\leq n}\right)).$$

when processing the data in *N* steps where $X_{<n}=\left(X_{1},\cdots ,X_{n-1}\right)$ and $X_{\leq n}=\left(X_{1},\cdots ,X_{n}\right)$. Thus, given that the equilibrium free-energy difference ΔF is a state function independent of the path (that means independent of whether data are processed all in one go or in small chunks), a system acquiring data slowly will have a reduced surprise S and therefore have less dissipation $U^{\mathrm{diss}}$.

**Figure 2:**
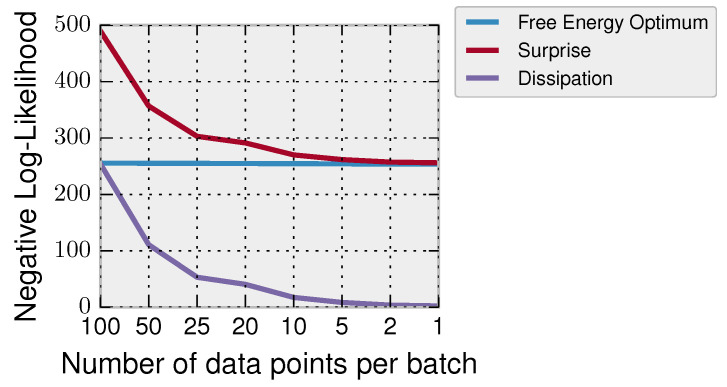
Surprise, dissipation and free energy optimum as a function of the number of data points per batch in a Bayesian inference task. When the decision-maker processes all the data in one step, it has maximum surprise and dissipation. However, when incorporating the data slowly, the surprise and dissipation are humble. The free energy optimum is only a function of the data independent of how they are incorporated.

In Figure 2, we show how the number of data chunks has an effect on the overall surprise and dissipation. In particular, we have a dataset $D=(x_{1},\cdots ,x_{T})$ consisting of T=100 data points Gaussian distributed $x∼N(x;\mu_{d}=5,\sigma_{d}^{2}=4)$ that we divide into batches of different sizes b∈{100,50,25,20,10,5,2,1}. The decision-maker has prior belief $p_{0}\left(\theta \right)$ about the mean $\theta =\mu_{d}$ and incorporates the data of every batch of data according to Bayes’ rule until all the data are incorporated. In general, the Bayesian learner processes the data in T/b steps; for example in the case of b=100, all data are processed at once (having thus high surprise), and in the case of b=1, it incorporates the data in *T* updates with an overall smaller surprise. In Figure 2, we show for different batch sizes the free energy optimum $\Delta F=\log\int p_{0}\left(\theta \right)p\left(D|\theta \right)$, the surprise S and the dissipation $U^{\mathrm{diss}}=\Delta F-S$. It can be seen that when acquiring the data in small chunks, the surprise of the decision-maker and the dissipation are lower.

### 3.4. Dissipation and Learning Hysteresis

A common paradigm to study how humans learn is through adaptation tasks where subjects are exposed to changes in an environmental variable that they can counteract by changing an internal variable. Sensorimotor adaptation in humans has been extensively studied in these error-based paradigms, for example where subjects have to adapt their hand position (internal variable) to change a virtual end effector position represented by a dot on a screen (external variable).

Consider a utility function $U_{v}\left(x\right)=-{\left(x-\mu_{v}\right)}^{2}$. For v=0, we determine the prior behaviour of a decision-maker with $p_{0}\left(x\right)=\frac{e^{\beta U_{0}\left(x\right)}}{Z}$. Initially, the decision-maker obtains an average utility of ${\langle U_{0}\rangle }_{p_{0}}$, which corresponds to zero mismatch between the decision-maker and the environmental variable. A change of the environmental variable to v=1 effectively changes the utility function to $U_{1}\left(x\right)=-{\left(x-\mu_{1}\right)}^{2}$, making $p_{0}$ non-optimal. This forces the decision-maker to reduce error adapting to the environmental variable by changing its probability distribution over his/her actions. When fully adapted to the new environment, the decision-maker again makes no errors (other than the errors due to motor noise). We illustrate this adaptation paradigm with a decision-maker that adapts according to the Metropolis–Hastings algorithm, which follows Markovian dynamics [52].

#### Crooks Theorem and Hysteresis Effects in Adaptation Tasks

Limited adaptation capabilities not only have an effect on the amount of obtained utility through the second law for decision-making $U^{\mathrm{net}}\leq \Delta F$, but also induce a time asymmetry in sequential decision-making processes. Hysteresis loops are a typical example of this asymmetry. Hysteresis is the phenomenon in which the path followed by a system due to an external perturbation, e.g., from state *A* to *B*, is not the same as the path followed in the reverse perturbation, e.g., from state *B* to *A*. When the system follows the same path for the forward perturbation and for the reverse perturbation, we say that the process is time symmetric (and therefore, it is not subject to hysteresis effects).

**Figure 3:**
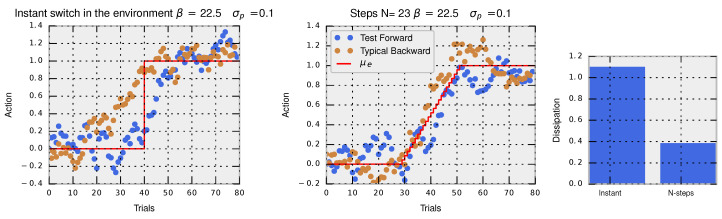
Trajectories of actions from the Metropolis–Hastings algorithm with β=22.5 and the proposed standard deviation $\sigma_{p}=0.1$ in a forward (blue) or backward (brown) protocol for an instant change in the environment (first panel) and for a slow change in the environment (second panel). In both cases, the total change in the environment is $\mu_{0}=0$ to $\mu_{1}=1$. The last panels shows the dissipation for the forward protocol (blue) in both the instant or the slow change in the environment. The difference in probability densities of forward and backward trajectories relates directly to dissipation and to hysteresis effects.

In the two left panels of Figure 3, we show a simulated trajectory of actions composed of 80 trials for an adaptation task using the Metropolis–Hastings algorithm with β=22.5, a Gaussian proposal $g\left(x^{\prime }|x\right)=N\left(x^{\prime };\mu =x,\sigma_{p}=0.1\right)$ and acceptance criterion $\alpha \left(x^{\prime }|x\right)=\min\left(\frac{e^{\beta U(x^{\prime })}g\left(x|x^{\prime }\right)}{e^{\beta U(x)}g\left(x^{\prime }|x\right)},1\right)$, when changing the environmental variable from $\mu_{0}=0.0$ to $\mu_{1}=1.0$. In blue, we show the trajectory for the forward-in-time perturbation, which converges after a few dozen trials to the new equilibrium. In brown, we show the trajectory for the reversed perturbation where the process starts with the last trial (80) and ends with the initial trial (0). In the left panel, the perturbation is made instantaneously in one step at Trial 40 and in the right panel in multiple steps (N=23). The hysteresis effect is clearly seen in the instantaneous perturbation where the path of actions followed by the decision-maker in the forward perturbation is clearly different from a typical trajectory of actions taken when applying the reversed perturbation. When the perturbation is made in multiple steps, both typical backward and typical forward trajectories become more similar denoting a smaller hysteresis effect. In this way, hysteresis effects are tightly connected to the concept of dissipation.

Dissipation and the ratio between forward and backward probabilities of trajectories of actions correspond exactly to the Crooks theorem for decision-making:

$$\frac{p(x)}{p^{†}\left(x\right)}=e^{\beta U^{\mathrm{diss}}\left(x\right)}.$$

The probability of observing a trajectory of accepted actions $x=(x_{0},x_{1},\cdots x_{T})$ for the Metropolis–Hastings algorithm is easily computed with $p\left(x\right)=p\left(x_{0}\right)\prod_{t=1}^{T}g\left(x_{t}|x_{t-1}\right)\alpha \left(x_{t}|x_{t-1}\right)$. Similarly, the probability of observing the same trajectory in the backward protocol is $p\left(x^{†}\right)=p_{\mathrm{eq}}\left(x_{T}\right)\prod_{t=1}^{T}g\left(x_{T-t}|x_{T-t+1}\right)\alpha \left(x_{T-t}|x_{T-t+1}\right)$. The dissipated utility is $U^{\mathrm{diss}}=\Delta F-U_{\mathrm{tot}}$ where the free energy difference is computed between the final $p_{1}\left(x\right)=\frac{1}{Z}e^{\beta U_{1}\left(x\right)}$ and initial equilibrium distributions $p_{0}\left(x\right)=\frac{1}{Z}e^{\beta U_{0}\left(x\right)}$, and the total utility gained $U_{\mathrm{tot}}$ is the sum of the utilities $\Delta U(x,t_{n}\to t_{n+1})$ at each environmental change at time $t_{n}$. In the third panel of Figure 3, we show that the protocol with the instantaneous perturbation has higher dissipation (related to higher hysteresis) compared to the protocol with multiple small perturbations.

## 4. Generalized Non-Equilibrium Thermodynamics for Decision-Making with Deliberation

So far, we have studied decision-makers that were forced to select an action with no opportunity to respond to a change in the utility function. This could correspond, for example, to a scenario of trial-and-error learning, where the best available strategy is the prior strategy adapted to the environment before the utility changed. However, this restriction may not always be suitable. Consider for example a chess player that is shown a particular board configuration (corresponding to a change in utility) and now has a certain amount of time to decide on the next move. Similarly, consider the two introductory examples in Section 3, where we allow a sampling algorithm to run for a certain number of steps, and then, we stop and evaluate the action after the algorithm has adapted to the new utility. In general, such deliberation processes are expensive, and we assume in the following that the Kullback–Leibler divergence is an appropriate measure of this computational expense, as outlined in the Introduction.

In the following, we consider again decision-makers facing a sequence of decision problems expressed by the utility changes $\Delta U\left(x,t_{0}\to t_{1}\right),\cdots ,\Delta U\left(x,t_{N-1}\to t_{N}\right)$. In contrast to the previous section where decision-makers had to decide before they could adapt to the utility change, decision-makers that deliberate select their action $x_{n}$ after they have (partially) adapted to the utility change:

$$\Delta U\left(x_{n},t_{n-1}\to t_{n}\right)=U\left(x_{n},t_{n}\right)-U\left(x_{n},t_{n-1}\right).$$

Using this notation, we are able to summarize the decision-maker’s choice by a vector $x:=(x_{0},\ldots ,x_{N})$ and characterize its behaviour by the probability $p\left(x\right):=p\left(x_{0}|t_{0}\right)\prod_{n=1}^{N}p\left(x_{n}|x_{n-1},t_{n}\right)$ with $p\left(x_{0}|t_{0}\right)=p_{0}\left(x_{0}\right)$, assuming that the initial strategy is a bounded rational equilibrium strategy. Note that in the deliberation scenario, the initial state $x_{0}$ does not constitute a decision, but instead, we include the last decision $x_{N}$.

This setup is illustrated again in Figure 1 (right column) for a one-step decision problem $\Delta U(x,t_{0}\to t_{1})$ with behaviour vector $x=(x_{0},x_{1})$ and with an instantaneous change in the environment occurring at time $t_{0}$. In the deliberation scenario, the utility is determined after the deliberation time. During deliberation, the decision-maker has changed the strategy distribution from $p_{0}^{\mathrm{eq}}\left(x\right)$ to a non-equilibrium distribution $\tilde{p}\left(x\right)$ (for example, the distribution (6) in the rejection sampling scheme) spending in the process a certain amount of resources and achieving an average net utility of $U^{\mathrm{net}}=\Delta F\left[\tilde{p}\left(x\right)\right]$ according to Equation (3). In this case, the behaviour vector is $x=(x_{0},x_{1})$ with $x_{0}$ ignored and $x_{1}∼\tilde{p}\left(x\right)$. In such a scenario with a single decision problem, we define, in analogy with the previous section, the average dissipated utility as [24,53]:

(16) $$\begin{array}{cc} U^{\mathrm{diss}} & :=\Delta F-U^{\mathrm{net}}\\ & =\frac{1}{\beta }D_{\mathrm{KL}}\left(\tilde{p}\left(x\right)\|p_{1}^{\mathrm{eq}}\left(x\right)\right). \end{array}$$

See Appendix for a derivation of (16) from (9). It readily follows from the positivity of the relative entropy $D_{\mathrm{KL}}\left(p\|q\right)\geq 0$ that:

(17) $$U^{\mathrm{net}}\leq \Delta F$$

with equality when $\tilde{p}\left(x\right)=p_{1}^{\mathrm{eq}}\left(x\right)$. In the case of the rejection sampling decision-maker of Equation (6), this would correspond to an infinite amount of samples k→∞. The inequality (17) shows that we cannot obtain more utility than the equilibrium free energy difference.

Let us now look at the general case. In contrast to an agent without deliberation capabilities, an agent that deliberates will be able to act according to a different distribution than the prior strategy. This means that when facing the utility change $\Delta U(x,t_{n-1}\to t_{n})$ at time $t_{n}$, the agent chooses the action $x_{n}$ sampled from the posterior strategy, contrary to an agent without deliberation that chooses $x_{n-1}$ sampled from the prior strategy. The deliberation process incurs a computational cost that is measured (in a similar fashion to stochastic thermodynamics [54] and previous formulations of bounded rationality given in the introduction) with the difference between the conditional stochastic entropies from prior to posterior:

$$s\left(x_{n}|x_{n-1},t_{n}\right)-s\left(x_{n}|x_{n-1},t_{n-1}\right):=-\log\frac{p(x_{n}|x_{n-1},t_{n})}{p(x_{n}|x_{n-1},t_{n-1})}.$$

Note that the prior distribution $p(x_{n}|x_{n-1},t_{n-1})$ is the previous posterior distribution evaluated at $x_{n}$ instead of $x_{n-1}$. Basically, this measures the change in probability from prior behaviour to posterior behaviour of the newly chosen action $x_{n}$.

Taking into account the computational cost of deliberation, we define the net utility of action $x_{n}$ due to a change in the environment as

$$u\left(x_{n},t_{n-1}\to t_{n}\right)=\Delta U\left(x_{n},t_{n-1}\to t_{n}\right)-\frac{1}{\beta }\log\frac{p(x_{n}|x_{n-1},t_{n})}{p(x_{n}|x_{n-1},t_{n-1})},$$

which generalizes the concept of work from the previous section. The expected change in net utility is the objective function that the decision-maker optimizes at each time step. The total net utility $U^{\mathrm{net}}\left(x\right)=\sum_{n=1}^{N}u\left(x_{n},t_{n-1}\to t_{n}\right)$ takes the form of a non-equilibrium free energy:

(18) $$U^{\mathrm{net}}\left(x\right)=\sum_{n=1}^{N}\Delta U\left(x_{n},t_{n-1}\to t_{n}\right)-\frac{1}{\beta }\sum_{n=1}^{N}\log\frac{p(x_{n}|x_{n-1},t_{n})}{p(x_{n}|x_{n-1},t_{n-1})}.$$

at the trajectory level. Similarly to Equation (8), the first law for decision-making with deliberation costs is:

$$U^{\mathrm{net}}=\Delta F-U^{\mathrm{diss}}$$

and states that the total net utility $U^{\mathrm{net}}={\langle U^{\mathrm{net}}\left(x\right)\rangle }_{p(x)}$ is the difference between the bounded optimal utility (following the equilibrium strategy with precision β) expressed by the equilibrium free energy difference ΔF and the dissipated utility $U^{\mathrm{diss}}$. The dissipation:

(19) $$U^{\mathrm{diss}}\left(x\right):=\Delta F-U^{\mathrm{net}}\left(x\right)$$

measures the amount of utility loss if the decision-maker’s plan does not manage to produce an action from the equilibrium distribution, for example due to the lack of time for deliberation. However, a decision-maker with infinite deliberation time will not have this problem and therefore will not dissipate by wasting utility.

To investigate the counterpart of the second law, we need to determine whether $U^{\mathrm{diss}}\geq 0$ holds. This can be achieved, for example, by first deriving the counterpart of the Crooks fluctuation theorem or the counterpart of the Jarzynski equation with subsequent application of Jensen’s inequality. In the following two theorems, we assume that the decision-makers satisfy the detailed balance condition. The detailed balance condition ensures two important characteristics. First, the stochastic process reaches equilibrium, and second, it ensures time-reversibility when in equilibrium. In a decision-making scenario, this translates into the following. First, when given enough computation time, the decision-makers manage to sample actions from the correct equilibrium distributions. Second, ideal decision-makers in equilibrium should not produce any entropy, which is exactly what happens if detailed balance is satisfied.

**Theorem** **1.** *Crook’sfluctuation theorem for decision-making with deliberation costs states that:*

(20) $$\frac{p(x)}{p^{†}\left(x\right)}=e^{\beta U^{\mathrm{diss}}\left(x\right)}$$

*where the dissipated utility of a particular trajectory is $U^{\mathrm{diss}}\left(x\right)=\Delta F-U^{\mathrm{net}}\left(x\right)$ as defined in Equation (18) and the probability of the trajectory using the backward protocol is $p^{†}\left(x\right)=p^{†}\left(x_{0}|x_{1},t_{0}\right)$$p^{†}\left(x_{1}|x_{2},t_{1}\right)\cdots p^{†}\left(x_{N}|t_{N}\right)$ for N decision problems starting at time $t_{N}$ and going backwards up to $t_{0}$. For the relation to be valid, we must assume that the starting distribution in the backward process is also in equilibrium, $p\left(x_{N}|t_{N}\right)\propto e^{\beta U(x_{N},t_{N})}$.*

**Proof.**  Here, we derive the relationship between reversibility and dissipation.

$$\begin{array}{cc} \frac{p(x)}{p^{†}\left(x\right)} & =\frac{p\left(x_{0}|t_{0}\right)p\left(x_{1}|x_{0},t_{1}\right)\cdots p\left(x_{N}|x_{N-1},t_{N}\right)}{p^{†}\left(x_{0}|x_{1},t_{0}\right)p^{†}\left(x_{1}|x_{2},t_{1}\right)\cdots p^{†}\left(x_{N}|t_{N}\right)}\\ & =\frac{e^{\beta U(x_{0},t_{0})}}{Z_{0}}\frac{1}{e^{\beta U(x_{0},t_{0})}}\frac{p(x_{1}|x_{0},t_{1})}{p(x_{1}|x_{0},t_{0})}\frac{e^{\beta U(x_{1},t_{0})}}{e^{\beta U(x_{1},t_{1})}}\cdots \frac{p(x_{N}|x_{N-1},t_{N})}{p(x_{N}|x_{N-1},t_{N-1})}\frac{e^{\beta U(x_{N},t_{N-1})}}{e^{\beta U(x_{N},t_{N})}}Z_{N}\\ & =\frac{Z_{N}}{Z_{0}}e^{\beta \frac{1}{\beta }\log\frac{p(x_{1}|x_{0},t_{1})}{p(x_{1}|x_{0},t_{0})}}e^{-\beta \Delta U(x_{1},t_{0}\to t_{1})}\cdots e^{\beta \frac{1}{\beta }\log\frac{p(x_{N}|x_{N-1},t_{N})}{p(x_{N}|x_{N-1},t_{N-1})}}e^{-\beta \Delta U(x_{N},t_{N-1}\to t_{N})}\\ & =e^{\beta \Delta F-\beta U^{\mathrm{net}}\left(x\right)}=e^{\beta U^{\mathrm{diss}}\left(x\right)} \end{array}$$

where in the second line, we have substituted $p^{†}\left(x_{n-1}|x_{n},t_{n-1}\right)$ using the identity:

$$p^{†}\left(x_{n-1}|x_{n},t_{n-1}\right)=\frac{e^{\beta U(x_{n-1},t_{n-1})}}{e^{\beta U(x_{n},t_{n-1})}}p\left(x_{n}|x_{n-1},t_{n-1}\right)$$

from detailed balance, and we assumed the initial distribution to be in equilibrium $p\left(x_{0}|t_{0}\right)=\frac{e^{\beta U(x_{0},t_{0})}}{Z_{0}}$ and that in the backward process the decision-maker starts also using the equilibrium strategy $p^{†}\left(x_{N}|t_{N}\right)=\frac{1}{Z_{N}}e^{\beta U(x_{N},t_{N})}$. In the third line, we cancel out terms and apply the following two equalities $\frac{p(x_{n}|x_{n-1},t_{n})}{p(x_{n}|x_{n-1},t_{n-1})}=e^{\beta \frac{1}{\beta }\log\frac{p(x_{n}|x_{n-1},t_{n})}{p(x_{n}|x_{n-1},t_{n-1})}}$ and $\Delta U\left(x_{n},t_{n-1}\to t_{n}\right)=U\left(x_{n},t_{n}\right)-U\left(x_{n},t_{n-1}\right)$. Finally, in the last line, we employ the definition of the net utility in Equation (18) and $\frac{Z_{N}}{Z_{0}}=e^{\beta \Delta F}$. ☐

Although at first sight, Equation (20) looks the same as the previous Crooks’ relation for the no-deliberation case (12), it is not the same. Here, the net utility is defined by Equation (18), which takes into account both the gain in utility and the computational costs of deliberating.

**Theorem** **2.** *The Jarzynski equality for decision-making with deliberation costs states that:*

(21) $${\langle e^{\beta U^{\mathrm{net}}\left(x\right)}\rangle }_{p(x)}=e^{\beta \Delta F}.$$

**Proof.**  $$\begin{array}{c} {\langle \exp\left(\beta \sum_{n=1}^{N}\left[\Delta U\left(x_{n},t_{n-1}\to t_{n}\right)-\frac{1}{\beta }\log\frac{p(x_{n}|t_{n},x_{n-1})}{p(x_{n}|t_{n-1},x_{n-1})}\right]\right)\rangle }_{p(x)}=\\ \overset{\left(1.\right)}{=}\sum_{x_{0},\;x_{n},\cdots x_{N}}p\left(x_{0}|t_{0}\right)\prod_{n=1}^{N}p\left(x_{n}|t_{n},x_{n-1}\right)\prod_{n=1}^{N}\frac{\exp(\beta U\left(x_{n},t_{n}\right))}{\exp(\beta U\left(x_{n},t_{n-1}\right))}\prod_{n=1}^{N}\frac{p(x_{n}|t_{n-1},x_{n-1})}{p(x_{n}|t_{n},x_{n-1})}\\ \overset{\left(2.\right)}{=}\sum_{x_{0},\cdots x_{n},\cdots x_{N}}p\left(x_{0}|t_{0}\right)\frac{\exp(\beta U\left(x_{1},t_{1}\right))}{\exp(\beta U\left(x_{1},t_{0}\right))}\prod_{n=2}^{N}\frac{\exp(\beta U\left(x_{n},t_{n}\right))}{\exp(\beta U\left(x_{n},t_{n-1}\right))}p\left(x_{1}|t_{0},x_{0}\right)\prod_{n=2}^{N}p\left(x_{n}|t_{n-1},x_{n-1}\right)\\ \overset{\left(3.\right)}{=}\frac{1}{Z_{0}}\sum_{x_{1}\cdots x_{n},\cdots x_{N}}\exp\left(\beta U\left(x_{1},t_{1}\right)\right)\prod_{n=2}^{N}\frac{\exp(\beta U\left(x_{n},t_{n}\right))}{\exp(\beta U\left(x_{n},t_{n-1}\right))}\prod_{n=2}^{N}p\left(x_{n}|t_{n-1},x_{n-1}\right)\\ \overset{\left(4.\right)}{=}=\frac{1}{Z_{0}}\sum_{x_{2}\cdots x_{n},\cdots x_{N}}\prod_{n=2}^{N}\frac{\exp(\beta U\left(x_{n},t_{n}\right))}{\exp(\beta U\left(x_{n},t_{n-1}\right))}\prod_{n=3}^{N}p\left(x_{n}|t_{n-1},x_{n-1}\right)\underset{=\exp(\beta U\left(x_{2},t_{1}\right))(\mathrm{Detailed}\;\mathrm{Balance})}{\underset{︸}{\sum_{x_{1}}\exp\left(\beta U\left(x_{1},t_{1}\right)\right)p\left(x_{2}|t_{1},x_{1}\right)}}\\ \overset{\left(5.\right)}{=}\frac{1}{Z_{0}}\sum_{x_{N}}\exp\left(\beta U\left(x_{N},t_{N}\right)\right)=\frac{Z_{N}}{Z_{0}}=e^{\beta \Delta F} \end{array}$$

In (1.), we unfold the expression and exploit the equality $e^{\log p+\log q}=pq$ for the summation inside the exponential. In (2.), we cancel the trajectory probabilities $\prod_{n=1}^{N}p\left(x_{n}|t_{n},x_{n-1}\right)$ and then take one term out of the two remaining products. In (3.), first, we use the equivalence $\exp\left(\beta U\left(x_{1},t_{0}\right)\right)=Z_{0}p_{\mathrm{eq}}\left(x_{1}|t_{0}\right)$ (because at time $t_{0}$, the decision-maker is acting according to the equilibrium distribution) that allows us to cancel with $p\left(x_{1}|t_{0},x_{0}\right)=p_{\mathrm{eq}}\left(x_{1}|t_{0}\right)$, and second, we sum over $x_{0}$ with the only term that depends on it being $p(x_{0}|t_{0})$. In (4.), we take one term of the second product and perform the sum over $x_{1}$ to obtain by detailed balance $\exp(\beta U\left(x_{2},t_{1}\right))$ that will allow us to cancel with the term in the denominator of the first product. We perform Steps (3.) and (4.) repeatedly until obtaining the last equivalence that proves the theorem.

Again, we note that the previously-proven Jarzynski relation from Equation (21) is not the same equation as in the no-deliberation case (13). In the deliberation case, the definition of the net utility is different and takes into account both the utility gain and the computational cost of deliberating.

We can now state the second law of decision-making with deliberation costs as:

(22) $${\langle U^{\mathrm{diss}}\left(x\right)\rangle }_{p(x)}=\frac{1}{\beta }D_{\mathrm{KL}}\left(p\left(x\right)\|p^{†}\left(x\right)\right)\geq 0$$

from Equation (20) by rearranging and taking expectations. The same inequality can be obtained from Equation (21) by applying Jensen’s inequality $\langle \exp x\rangle \geq \exp\langle x\rangle$ to recover ${\langle U^{\mathrm{net}}\left(x\right)\rangle }_{p(x)}\leq \Delta F$. Equation (21) connects finite with infinite time decision-making. That is, there is a relation between the equilibrium free-energy differences that is the maximum attainable net utility with unlimited computation time and the net utility obtained by decision-makers with limited computation time. In the next section, we will provide examples of how to use these relations to extract useful information from decision-making processes.

### 4.1. Examples

For the deliberation scenario, we illustrate the novel Jarzynski equality and Crooks theorem for decision-making in two decision-making scenario with clearly defined independent episodes: the first case is a discrete decision-making problem, and the second case is a continuous decision-making problem.

#### 4.1.1. Jarzynski and Crooks Relations for Episodic Decision-Making with Deliberation

**Figure 4:**
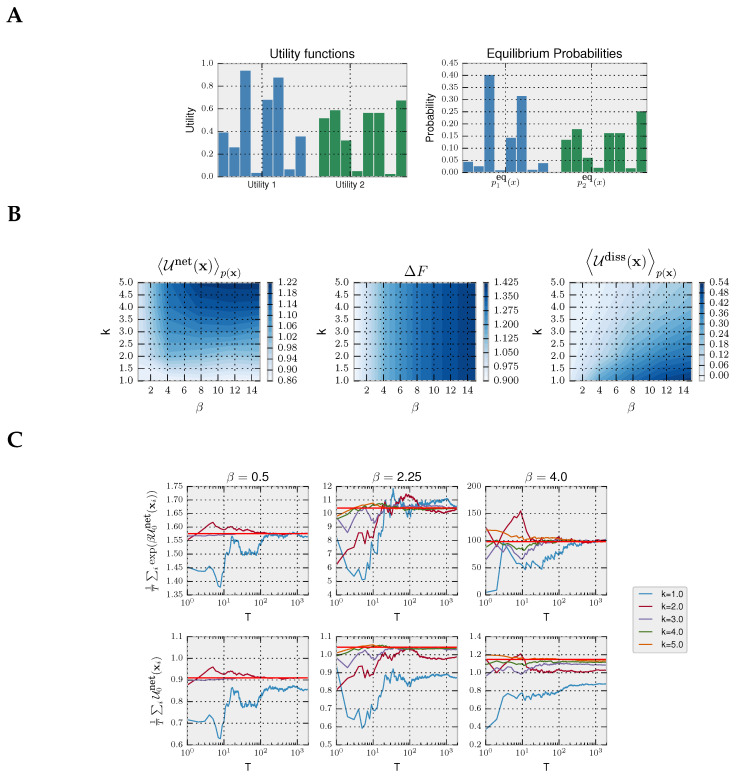
Episodic decision-making with deliberation. (**A**) Utility functions and equilibrium distributions for the two decision problems; (**B**) we show for different β and *k* (left) the average net utility, (middle) the free energy difference and (right) the average dissipated utility; (**C**) top panels: empirical averages approximating the Jarzynski expression in dependence of the number of trajectories *T* using different β and different number of available samples *k*; bottom panels: the associated expected net utility gain, which in the limit T→∞ is lower than the free energy difference (horizontal light red line).

Choice-reaction-time experiments aimed to study information-processing in humans typically consider episodic tasks consisting of many trials; see [55] for a recent example. Here, we take a variation of Hicks episodic task with discrete action space, commonly used in the decision-making literature. In our variation of Hicks task, the decision-maker is shown a set of eight light bulbs. Initially, all light bulbs are turned off. Upon stimulus presentation, all light bulbs are turned on with different light intensities (representing different utilities) for a limited amount of time in which the decision-maker must choose the brightest light associated with the highest utility. The choice task is repeated many times, each time with different light intensities. For simplicity, our example contains only two stimuli: compare Utility 1 and Utility 2 in Figure 4A. When given enough time, a decision-maker with prior $p_{0}\left(x\right)$ chooses its actions according to the equilibrium distribution from Equation (4), as illustrated in Figure 4A for the uniform prior $p_{0}\left(x\right)=\frac{1}{8}$ that we assume in our example. In this case, the precision β specifies how well the light intensities can be told apart by a bounded optimal decision-maker.

In Figure 4, we model a decision-maker using the rejection sampling algorithm with the most efficient aspiration level given by the maximum utility $\max_{x}\Delta U\left(x\right)$. In particular, we simulate the rejection sampling algorithm with a limited number of samples (parameterized by *k*), where the choice strategy is given by non-equilibrium probability distribution in Equation (6) from the Introduction, because we assume that a response has to be produced within a fixed amount of time.

In this kind of episodic task, the decision-maker always starts with the same prior $p_{0}\left(x\right)$ over the possible choices *x*. The probability of a trajectory of decisions x is defined as $p\left(x\right):=\prod_{n=1}^{N}p\left(x_{n}|t_{n}\right)$ for each episode *n*, and the net utility for a trajectory is:

$$U_{0}^{\mathrm{net}}\left(x\right):=\sum_{n=1}^{N}\left[\Delta U\left(x_{n},t_{n-1}\to t_{n}\right)-\frac{1}{\beta }\log\frac{p(x_{n}|t_{n})}{p_{0}\left(x_{n}\right)}\right].$$

Consequently, the equilibrium free energy is defined as $\Delta F:=\max_{\tilde{p}\left(x\right)}{\langle U_{0}^{\mathrm{net}}\left(x\right)\rangle }_{\tilde{p}\left(x\right)}$, which can also be decomposed into the sum of *N* independent equilibrium free energies $\Delta F=\sum_{n=1}^{N}{\langle \Delta U\left(x_{n},t_{n-1}\to t_{n}\right)-\frac{1}{\beta }\log\frac{p^{\mathrm{eq}}\left(x_{n}|t_{n}\right)}{p_{0}\left(x_{n}\right)}\rangle }_{p^{\mathrm{eq}}\left(x_{n}|t_{n}\right)}$ where:

$$p^{\mathrm{eq}}\left(x_{n}|t_{n}\right)=\frac{p_{0}\left(x_{n}\right)\exp\left(\beta \Delta U\left(x_{n},t_{n-1}\to t_{n}\right)\right)}{Z_{n}}$$

and the dissipated utility for a trajectory is $U^{\mathrm{diss}}\left(x\right):=\Delta F-U_{0}^{\mathrm{net}}\left(x\right)$.

We simulate trajectories with N=2 by sampling repeatedly from Equation (6). In the first panel of Figure 4B, we show that, as expected, the more samples *k* a decision-maker can afford, the higher the average net utility ${\langle U_{0}^{\mathrm{net}}\rangle }_{p(x)}$. In the second panel, it can be seen that the equilibrium free energy difference is invariant with respect to *k* and increases with higher precision β. Lastly, in the third panel, we plot the average dissipated utility ${\langle U^{\mathrm{diss}}\rangle }_{p(x)}$ that measures how much utility is lost due to the limited number of available samples. The highest dissipation occurs for high β and few samples *k* because such a high-precision decision-maker can potentially obtain high utility, but the limited amount of samples restrain it. In the following, we consider both a Jarzynski-like relation and a fluctuation theorem valid for a fixed prior.

##### Jarzynski Equality for Decision-Making with Fixed Prior $p_{0}$

For a fixed prior, it can readily be shown that the following relation is valid:

(23) $${\langle e^{\beta U_{0}^{\mathrm{net}}\left(x\right)}\rangle }_{p(x)}=e^{\beta \Delta F}.$$

To illustrate the validity of Equation (23), we simulated a decision-maker that faces *T* times the same two decision problems from Figure 4A. We can estimate the left-hand side of Equation (23) with the empirical average $\frac{1}{T}\sum_{i}\exp\left(\beta U_{0}^{\mathrm{net}}\left(x_{i}\right)\right)$ with the *T* trajectories of decisions, where $x_{i}∼p\left(x\right)$. In the top row of Figure 4C, we show the empirical average converging to exp(βΔF) (as expected by the law of large numbers) depending on the number of simulated trajectories *T* and precision β, empirically validating Equation (23). In the bottom row, we show how the second law for decision-making is fulfilled as the average net utility is less than the equilibrium free energy, thus satisfying the inequality (17).

##### Crooks’ Fluctuation Theorem for Decision-Making with Fixed Prior $p_{0}$

For the fixed prior, it can readily be shown that the following fluctuation relation holds:

(24) $$\frac{\tilde{p}\left(x\right)}{p^{\mathrm{eq}}\left(x\right)}=e^{\beta (\Delta F-U_{0}^{\mathrm{net}}\left(x\right))}=e^{\beta U^{\mathrm{diss}}\left(x\right)}$$

where $p^{\mathrm{eq}}\left(x\right):=\prod_{n=1}^{N}p^{\mathrm{eq}}\left(x_{n}|t_{n}\right)$ is the optimal equilibrium distribution over trajectories x. Note in this case that the probability distribution of the backward process $p^{†}\left(x\right)$ coincides with the optimal equilibrium distribution $p^{†}\left(x\right)=p^{\mathrm{eq}}\left(x\right)$ because of the independence of the decision problems. More specifically, the original Crooks theorem for decision-making from Equation (20) is valid only when the backward process starts in equilibrium. In our episodic task, all decision problems are independent, which makes the starting equilibrium distributions for all the backward processes coincide with the posterior equilibrium distributions of the forward process.

The fluctuation relation (24) for episodic tasks adopts a different meaning than the conventional relation. Specifically, the ratio between probabilities is now between the probability of observing a trajectory of actions when having finite time to make a decision (a sequence of non-equilibrium probabilities) and the probability of observing the same trajectory when having infinite time (a sequence of equilibrium probabilities). This ratio is governed by the exponential of the dissipated utility $U^{\mathrm{diss}}\left(x\right)$ similarly to the original Crooks equation.

Equation (24) can be rewritten by re-arranging the terms and averaging over p(x) as

$$\frac{1}{\beta }D_{\mathrm{KL}}\left(p\left(x\right)\|p^{\mathrm{eq}}\left(x\right)\right)={\langle U^{\mathrm{diss}}\left(x\right)\rangle }_{p(x)}.$$

Consequently, we see that purely from the trajectories of actions, we can obtain the average dissipated utility. We can test this relation in human experiments by comparing the trajectories of actions in two different conditions, first when having finite time and second when having as much time as needed. Then, from the probabilities of action trajectories, we can extract the average dissipated utility.

#### 4.1.2. Jarzynski and Crooks Relations for Deliberating Continuous Decisions

Since many decision tasks take place in the continuous domain (for example, sensorimotor tasks), we now consider continuous state space problems. In particular, we repeat the same analysis as in the previous section by validating our Jarzynski equation, but this time in the continuous domain. Moreover, in this example, we allow for adaptive changes in the prior, such that the prior in one trial is equal to the posterior of the previous trial. In the following, we model decision-making as a diffusion process with Langevin dynamics that stops after a certain time *t* and emits an action *x*. The diffusion process uses gradient information to find the optimum utility and will converge to an equilibrium distribution for t→∞. In our example, we will employ quadratic utility functions that allow for a closed form solution of the non-equilibrium probability density that changes over time.

Let x(t)∈R be the dynamics of computation that a decision-maker carries out when deliberating. The differential equation that describes the dynamics is:

(25) $$\frac{\partial x}{\partial t}=\alpha \frac{\partial U(x)}{\partial x}+\alpha \xi \left(t\right)$$

where ξ(t) is white Gaussian noise with mean 〈ξ(t)〉=0 and correlation $\langle \xi \left(t\right)\xi \left(t^{\prime }\right)\rangle =2D\delta \left(t-t^{\prime }\right)$. Note that Equation (25) is closely related to learning algorithms that use gradient information such as Stochastic Gradient Descent (SGD). These algorithms find the minimum of a cost function by taking steps in the state space in the opposite direction of the gradient. Here, we see that the learning rate corresponds to the parameter α, which, in contrast with plain GD, not only multiplies the gradient, but also the noise term.

Equation (25) gives the dynamics of the decision-making process in terms of a stochastic differential equation, which can equivalently be expressed by the evolution of the probability p(x,t) described by the Fokker–Planck equation [56]:

(26) $$\frac{\partial p(x,t)}{\partial t}=-\alpha p\left(x,t\right)\frac{\partial^{2}U\left(x\right)}{\partial x^{2}}-\alpha \frac{\partial U(x)}{\partial x}\frac{\partial p(x,t)}{\partial x}+D\alpha^{2}\frac{\partial^{2}p\left(x,t\right)}{\partial x^{2}}.$$

In order to compute the net utility, we need the probability of the non-equilibrium distribution up to a desired time *t*; thus, we need to solve the Fokker–Planck equation. For quadratic utility functions $U_{y}\left(x\right)=-\left(a_{y}x^{2}+b_{y}x\right)$ with coefficients $a_{y}$ and $b_{y}$ for environment *y* and initial Gaussian distribution with mean $\mu_{0}$ and variance $\sigma_{0}^{2}$, the solution is (see Appendix):

(27) $$p\left(x,t\right)=\frac{1}{\sqrt{2\pi \sigma^{2}\left(t\right)}}e^{\frac{-{\left(x-\mu \left(t\right)\right)}^{2}}{2\sigma^{2}\left(t\right)}}$$

with:

$$\begin{array}{cc} \sigma^{2}\left(t\right) & =\frac{\alpha^{2}D}{2c}\left(1-e^{-2ct}\right)+\sigma_{0}^{2}e^{-2ct}\\ \mu (t) & =e^{-ct}\mu_{0}-\frac{b_{1}}{2a_{1}}\left(1-e^{-ct}\right) \end{array}$$

where $c=2\alpha a_{1}$, and we assumed that the prior strategy is Gaussian distributed with mean $\mu_{0}$ and variance $\sigma_{0}^{2}$. The precision parameter relates to the other parameters with the relation $\beta =\frac{2\alpha }{D}$, which means that the higher the α, the more we take into account the gradient leading to a higher β, and the lower the noise *D*, also the higher β.

**Figure 5:**
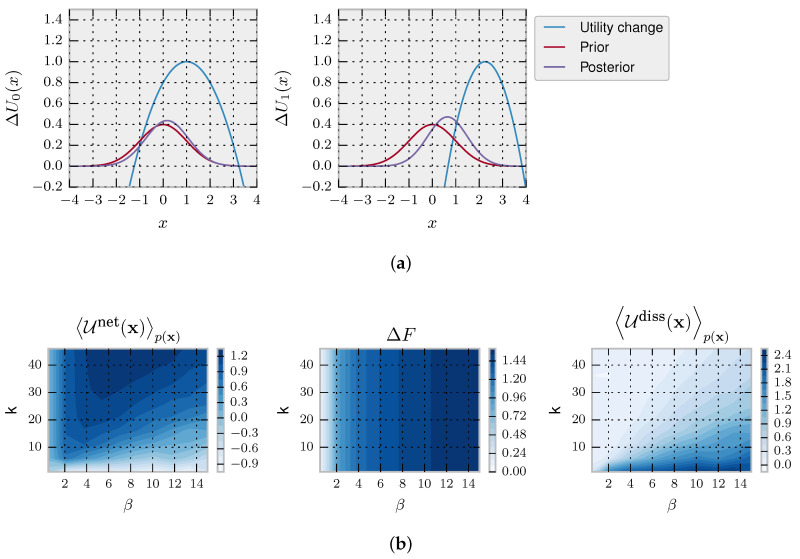

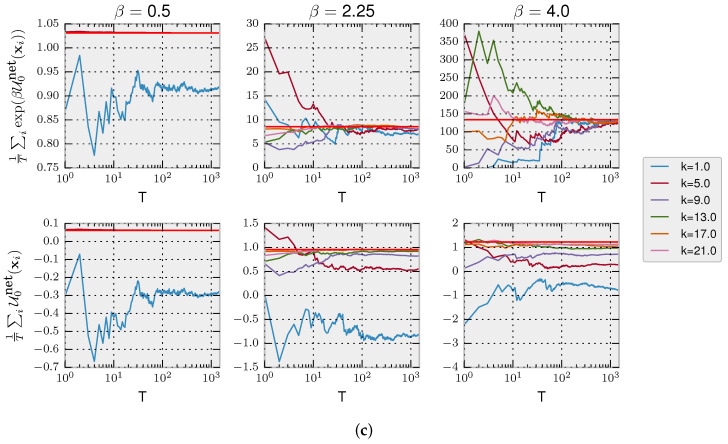
Langevin dynamics simulations. (**a**) In blue, the different utility changes $\Delta U_{1}$ and $\Delta U_{2}$, in red the prior $p_{0}$ and in purple the posterior for β=0.5; (**b**) We show for different β and time t=kΔt directly depending on *k*, (left) the average net utility, (middle) the free energy difference and (right) the average dissipated utility; (**c**) top panels: convergence of the empirical Jarzynski estimate depending on the number of trajectories *T* using different β and different numbers of update steps *k*. Bottom panels: the associated expected net utility gain, which in the limit T→∞ is lower than the free energy difference (horizontal light red line). With these simulations, we validate Equation (21).

Following a similar approach as in the previous section, we expose a decision-maker to two utility functions given by $U_{1}\left(x\right)=0.2x^{2}-0.4x-0.8$ and $U_{2}\left(x\right)=0.4x^{2}-1.8x+1.025$ shown in Figure 5A. The prior for the first utility is given by $\mu_{0}=0$ and $\sigma_{0}^{2}=1$. In Figure 5B, we show the net utility, equilibrium free-energy differences and dissipated utility (according to Equations (18) and (19)) for different values of β and number of steps *k*; corresponding to time t=kΔt in Equation (27) for a given reference Δt. In Figure 5C, we show the convergence of the Jarzynski term towards the true equilibrium free energy difference term depending on the number of trajectories to make the estimation. We can see on the bottom row that the second law for decision-making represented by the inequality (17) is fulfilled.

## 5. Discussion

In this paper, we highlighted the similarities between non-equilibrium thermodynamics and bounded rational decision-making in the case of agents that can deliberate before selecting an action and agents that cannot. Additionally, we derived a novel Jarzynski equality and a Crooks fluctuation theorem for decision-making scenarios with deliberation. We have shown how to use Jarzynski’s and Crooks’ equations in different scenarios to extract relevant variables of the decision-making process such as the equilibrium free energy difference, the average dissipated utility and the action-path probabilities for both equilibrium posterior distributions and distributions of the backward-in-time protocol. We have provided a number of examples for the no-deliberation and deliberation scenario, such as one-step lag dynamics, discrete choice tasks and continuous decision-making tasks that may be applicable both to cognitive and sensorimotor experiments [57].

In Section 3, we started out by directly translating physical non-equilibrium concepts to the decision-making domain in the case of decision-makers that cannot deliberate before acting and therefore lag behind changes in the utility landscape. In analogy to physical systems, we assumed that such decision-makers adapt to each utility change even though they are lagging behind, i.e., even after they have already chosen their action and there is no benefit of this adaptation at the current time step, but to improve their prior for the next choice. In physical systems, this does not constitute an issue, because there is a continuous adaptation to the energy gradient at every instant independent of how time is discretized. However, in the decision-making scenario, we assumed a single distinguished moment where the action is issued and the utility is evaluated. Therefore: Why should such decision-makers adapt at all after the action has been selected? Following the argument of no-free lunch theorems, there would be no benefit in adapting to arbitrary changes. Having a closer look at our examples in Section 3.3, it becomes evident that we implicitly assumed that the utility changes in each step were small, so there is a benefit in adapting the prior for the next trial. Such assumptions are typically made in learning scenarios, for example the i.i.d. assumption for inference problems or assumptions that utility changes in each time step are limited to a finite interval in decision-making problems. However, none of the non-equilibrium relations we discussed necessarily assume small utility changes. It should therefore be noted that, while the discussed non-equilibrium relations hold for arbitrary utility changes, in the context of non-deliberative decision-making, we would have to make additional assumptions such that utility changes in each step are small and can accumulate so that adaptation is beneficial. Importantly, the appropriateness of adaptation is not an issue when we assume a deliberation process where adaptation occurs before emitting an action, as there is a direct benefit of adaptation in the current trial. This is the general decision problem discussed in Section 4.

While we have considered mainly non-sequential decision-making problems here for simplicity, the same formalism could also be applied to sequential decision-making problems. In that case, one would replace the notion that an action corresponds to a discrete or continuous state *x* with the notion that an action might consist of choosing an entire trajectory $x_{1:\tau }$. In this case also, the utility $U(x_{1:\tau },t)$ would be defined over trajectories, and these utilities would change over episodes *t*. Again, one would have to assume that the utility function does not change while the trajectory $x_{1:\tau }$ is generated. This corresponds to the fact that we assume that the utility is constant for each single episode *t* (cf. Figure 1), while the deliberative decision-maker can, as it were, sample the new utility function before emitting an action. An example would be finding a trajectory for a pendulum swing-up or a sequence of actions to navigate a maze. A path integral controller [58] would for example exactly produce such trajectories. A deliberative decision-maker would sample many such trajectories until time is up and one trajectory has to be selected, then the utility changes again, and the path integral controller samples new trajectories that have a different shape in line with the new utility function. Our assumption that the temporal evolution of the utility function does not depend on the decision-maker’s action implies that consecutive episodes are independent and can have different utility functions, but the decision-maker can carry its prior from one episode over to the next.

Recently, there has been a renewed interest in modelling decision-making with computational constraints [59,60] both in the computer science and the neuroscience literature, where there is growing evidence that the human brain might exploit sampling [22,61,62,63,64,65] for approximate inference and decision-making [66,67]. Such sampling models have been used for example to explain anchoring biases in choice tasks, because MCMC has finite mixing times and therefore exhibits a dependence on the prior distribution [68,69]. In particular, the idea of using the (expected) relative entropy or the mutual information as a computational cost has been suggested several times in the literature [2,3,23,33,70,71,72]. In [33] and similarly in [20], the authors derive the relative entropy as a control cost from an information-theoretic point of view, under axioms of monotonicity and invariance under relabelling and decomposition. In other fields such as robotics, the relative entropy has also been used as a control cost [18,21,25,58,73,74] to regularize the behaviour of the controller by penalizing controls that are far from the uncontrolled dynamics of the system or to deal with model uncertainty [75]. Naturally, questions regarding the generality of entropic costs as information-processing costs and their potential relation to algorithmic space-time resource constraints carry over to the non-equilibrium scenario and remain a topic for future investigations.

So far, only very few studies have established connections between non-equilibrium thermodynamics and decision-making in the literature, even though non-equilibrium analysis might provide a promising way to relate mechanistic dynamical models to conceptually simpler utility-based models that are often employed as normative models. Jarzynski-like and Crooks-like relations have been noted in the economics literature in gambling scenarios [76] and when studying the arrow of time for decision-making [77,78]. We reported preliminary results for the one-step delayed decision-making in [79,80]. In the machine learning literature, generalized fluctuation theorems have recently been used in [81] to train artificial neural networks with efficient exploration. In general, fluctuation theorems and Jarzynski equalities allow one to estimate free energy differences, which are very important in decision-making because the free energy directly relates to the value function, which is a central concept in control and reinforcement learning. Fluctuation theorems typically make the assumption that the temperature parameter is constant (isothermal transformations) and that initial states are in equilibrium. In our paper, we also made these assumptions, which may limit the generality of our results. Loosening these restrictions (cf. for example [82,83]) might be an important next step for future investigations of non-equilibrium relations in the decision-making context.

Regarding the connection between predictive power and dissipation, [24] has found that non-predictive systems are also systems that are highly dissipative. In [24], the authors consider the effects of a stochastic driving signal *x* mediated by an energy function E(x,s) on the state *s* of a Markov system with fixed transition probability $p(s^{\prime }|s,x)$. They regard the Markov system as a computing device and study how much information the state *s* carries about the driving signal *x*. They find a fundamental relationship between dissipation (energy efficiency) and lack of predictive power. Their results concern non-equilibrium trajectories when *x* changes at every time point. The intuition is that when a system naturally moves in the direction of a changing energy landscape, then this is not only more efficient energetically, but it can also be interpreted in the sense that the system predicts the changing energy landscape. Once the system equilibrates, the energy landscape (i.e., the external variable x) does not change any more, and the mutual information between state and external variable xvanishes, as does the dissipation. Therefore, the equilibrium state is of no particular interest in this analysis. If one were to apply this framework to a decision-maker, the decision-maker would be represented by the system with the state *s*, and the driving signal *x* would be the input provided to the decision-maker. One important difference between [24] and our formulation is that in [24], the driving signal *x* is stochastic and is sampled from a stationary probability distribution, whereas in our formulation, we assume a fixed deterministic driving signal (the sequence of utility functions) without an underlying probability distribution. Assuming such a fixed input does prohibit an analysis in terms of mutual information between *s* and *x*. Nevertheless, it would be straightforward to allow for stochastic changes in the utility function also in our formulation, and the results of [24] would be applicable and complementary. While in [24], the equilibrium is of no particular interest, in our analysis, we are interested in the approach to equilibrium and in the resources spent on the way, that is the time that is spent during deliberating where the environment is assumed to be roughly constant, i.e., it does not change too much on the short time scale of deliberating, then the environment changes again, and the decision-maker can adapt to this change by deliberation (in contrast, in [24], the decision-maker follows a fixed dynamics and does not adapt).

In conclusion, the results presented here bring the fields of stochastic thermodynamics and decision-making closer together by studying decision-making systems as statistical systems just like in thermodynamics. In this analogy, the energy function in physics corresponds to the utility functions in decision-making. Importantly, the statistical ensembles of both decisions and physical states can be conceptualized as non-equilibrium ensembles that reach equilibrium after a finite time adaptation process.

## Appendix A. Dissipation for a One-Step Decision Problem

In the following, we derive Equation (16) from Equation (9) for a one-step decision problem. Let $x=(x_{0},x_{1})$. The probabilities p(x) of the forward trajectory are $p\left(x\right)=p_{0}^{\mathrm{eq}}\left(x_{0}\right)p\left(x_{1}|x_{0},t_{1}\right)$, and the probabilities $p^{†}\left(x\right)$ of the backward trajectory are $p^{†}\left(x\right)=p_{1}^{\mathrm{eq}}\left(x_{1}\right)p^{†}\left(x_{0}|x_{1},t_{0}\right)$. The detailed balance condition allows us to re-write $p^{†}\left(x_{0}|x_{1},t_{0}\right)$ as $p^{†}\left(x_{0}|x_{1},t_{0}\right)=\frac{e^{\beta U(x_{0},t_{0})}}{e^{\beta U(x_{1},t_{0})}}p\left(x_{1}|x_{0},t_{0}\right)$ with $e^{\beta U(x_{0},t_{0})}=Z_{0}p_{0}^{\mathrm{eq}}\left(x_{0}\right)$ and $e^{\beta U(x_{1},t_{0})}=Z_{0}p_{0}^{\mathrm{eq}}\left(x_{1}\right)$. With our notation in the deliberation scenario, $x_{1}$ is the decision, and $x_{0}$ is arbitrary and can be ignored. This effectively implies independence between $x_{0}$ and $x_{1}$, such that $p\left(x_{1}|x_{0},t_{1}\right)=p\left(x_{1}|t_{1}\right)$ and $p^{†}\left(x_{0}|x_{1},t_{0}\right)=\frac{p_{0}^{\mathrm{eq}}\left(x_{0}\right)}{p_{0}^{\mathrm{eq}}\left(x_{1}\right)}p\left(x_{1}|t_{0}\right)=p_{0}^{\mathrm{eq}}\left(x_{0}\right)$. Substituting the previous identities in the KL-divergence, we obtain:

$$\begin{array}{cc} \frac{1}{\beta }D_{\mathrm{KL}}\left(p\left(x\right)\|p^{†}\left(x\right)\right) & =\frac{1}{\beta }\sum_{x_{0},x_{1}}p_{0}^{\mathrm{eq}}\left(x_{0}\right)p\left(x_{1}|t_{1}\right)\log\frac{p_{0}^{\mathrm{eq}}\left(x_{0}\right)p\left(x_{1}|t_{1}\right)}{p_{1}^{\mathrm{eq}}\left(x_{1}\right)p_{0}^{\mathrm{eq}}\left(x_{0}\right)}\\ & =\frac{1}{\beta }D_{\mathrm{KL}}\left(p\left(x_{1}|t_{1}\right)\|p_{1}^{\mathrm{eq}}\left(x_{1}\right)\right)=\frac{1}{\beta }D_{\mathrm{KL}}\left(\tilde{p}\left(x\right)\|p_{1}^{\mathrm{eq}}\left(x\right)\right) \end{array}$$

where we have made the replacement $p\left(\cdot |t_{1}\right)=\tilde{p}\left(\cdot \right)$ to obtain the notation from Figure 1.

## Appendix B. Fokker-Planck Solution of Continuous Decision-Making Problem

A solution of the Fokker-Planck Equation (26) for known initial state $x_{0}$ can be found in [84]. Here, we sketch the solution when the initial state is Gaussian distributed.

Consider the following dynamics:

$$\frac{dx}{dt}=A\left(x,t\right)+B\left(x,t\right)\xi \left(t\right)$$

where $A\left(x,t\right)=\alpha \frac{\partial U_{1}}{\partial x}$, B(x,t)=α. When imposing a quadratic utility function:

$$U_{y}\left(x\right)=-\left(a_{y}x^{2}+b_{y}x\right)$$

for an environment indexed by y=1, the associated Fokker–Planck equation is

$$\frac{\partial P}{\partial t}=2\alpha a_{1}\frac{\partial }{\partial x}xP+\alpha b_{1}\frac{\partial }{\partial x}P+\alpha^{2}D\frac{\partial^{2}}{\partial x^{2}}P.$$

We will solve this equation by first taking the Fourier transform in the variable *x* and then solving by the method of characteristics. The Fourier transform is:

$$\begin{array}{cc} \frac{\partial \hat{P}}{\partial t}= & -cs\frac{\partial \hat{P}}{\partial s}-\alpha^{2}Ds^{2}\hat{P}+\alpha b_{1}is\hat{P}\\ = & -cs\frac{\partial \hat{P}}{\partial s}+\hat{P}\left(c_{2}is-\alpha^{2}Ds^{2}\right) \end{array}$$

where $c=2\alpha a_{1}$ and $c_{2}=\alpha b_{1}$. Now, applying the method of characteristics:

$$\frac{d\hat{P}}{dx}=\frac{\partial \hat{P}}{\partial s}\frac{ds}{dx}+\frac{\partial \hat{P}}{\partial t}\frac{dt}{dx}$$

we obtain that dt=dx, $s=s_{0}e^{ct}$, and applying these relations, we get:

$$\frac{d\hat{P}}{dx}=\frac{d\hat{P}}{dt}=\hat{P}\left(c_{2}is_{0}e^{ct}-\alpha^{2}Ds_{0}^{2}e^{2ct}\right)$$

Integrating over *t* between t=0 and $t=t^{\prime }$, we have that

$$\begin{array}{cc} \frac{d\hat{P}}{\hat{P}} & =dt\left(c_{2}is_{0}e^{ct}-\alpha^{2}Ds_{0}^{2}e^{2ct}\right)\\ \log\hat{P}{|}_{\hat{P}\left(s_{0},t=0\right)}^{\hat{P}\left(s,t^{\prime }\right)} & =\frac{c_{2}is_{0}}{c}e^{ct}-\frac{\alpha^{2}D}{2c}s_{0}^{2}e^{2ct}{|}_{t=0}^{t=t^{\prime }}. \end{array}$$

Assuming a Gaussian distribution as a boundary condition with mean $\mu_{0}$ and variance $\sigma_{0}^{2}$, the Fourier transform for the boundary is:

$$\hat{P}\left(s,t=0\right)=\exp\left\{-\frac{\sigma_{0}^{2}}{2}s_{0}^{2}-is_{0}\mu_{0}\right\}.$$

Then, the solution in frequency space is:

$$\begin{array}{cc} \hat{P}\left(s,t\right) & =\exp\left\{-\frac{\alpha^{2}D}{2c}s^{2}\left(1-e^{-2ct}\right)-\sigma_{0}^{2}s^{2}e^{-2ct}+is\frac{b_{1}}{2a_{1}}\left(1-e^{-ct}\right)-ise^{-ct}\right\}\\ & =\exp\left\{s^{2}f_{1}\left(t\right)-isf_{2}\left(t\right)\right\} \end{array}$$

with $f_{1}\left(t\right)=-\frac{\alpha^{2}D}{2c}\left(1-e^{-2ct}\right)-\frac{\sigma_{0}^{2}}{2}e^{-2ct}$ and $f_{2}\left(t\right)=e^{-ct}\mu_{0}-\frac{b_{1}}{2a_{1}}\left(1-e^{-ct}\right)$. Transforming back to the signal domain, we obtain:

$$\begin{array}{cc} \sigma^{2}\left(t\right) & =-2f_{1}\left(t\right)=\frac{\alpha^{2}D}{c}\left(1-e^{-2ct}\right)+\sigma_{0}^{2}e^{-2ct}\\ \mu (t) & =f_{2}\left(t\right)=e^{-ct}\mu_{0}-\frac{b_{1}}{2a_{1}}\left(1-e^{-ct}\right). \end{array}$$
